# SG-APSIC1171: A new approach of hand hygiene observation with focus on healthcare worker (HCW) category

**DOI:** 10.1017/ash.2023.48

**Published:** 2023-03-16

**Authors:** Wee Ling Tee, Dale Fisher, Cathrine Teo, Razali Bin Mahdi, Yvonne Lum

**Affiliations:** National University Hospital, Singapore; Singapore National University Hospital, Singapore; Singapore National University Hospital, Singapore; Singapore National University Hospital, Singapore; Singapore National University Hospital, Singapore

## Abstract

**Objectives:** The past hand hygiene (HH) compliance rate has indicated the low number of opportunities for some healthcare workers (HCWs) because the infection control liaison officer (ICLO) tended to capture opportunities from nurses who were available, despite the proportional allocation of opportunities per HCW type based on the World Health Organization (WHO) HH methodology. Therefore, HH compliance rates may not have accurately represented the specific HCW type, which may have affected the overall HH compliance rate. We sought to determine an accurate baseline of HH compliance rate with consistent number of opportunities across all HCW categories. **Methods:** HH auditors were ICLOs trained in HH observation by the infection control nurse (ICN) according to the WHO “My Five Moments of Hand Hygiene.” HH observations were conducted bimonthly with assigned areas focusing only on 1 HCW category for each session: nursing, medical, clinical support services, or environmental services. A briefing session was given on the day of observation, with the goal of collecting 20 opportunities per area with HCW focus during their peak activities. Direct feedback and positive reinforcement were given to HCWs after observations were completed. **Results:** A survey of 96 ICLOs indicated that observations based on HCW focus allowed them to capture more HH opportunities and concentrate on their observations. The new approach showed a significant increase in number of opportunities across all HCW categories that was more representative. We also successfully determined a new baseline for all HCW categories, with further breakdown of HCW type. **Conclusions:** A new methodology of HH observation with a focus on HCW category has resulted in more HH opportunities across all HCW categories and improved representation of the HH compliance rate.

